# Dose‐finding methods for Phase I clinical trials using pharmacokinetics in small populations

**DOI:** 10.1002/bimj.201600084

**Published:** 2017-03-21

**Authors:** Moreno Ursino, Sarah Zohar, Frederike Lentz, Corinne Alberti, Tim Friede, Nigel Stallard, Emmanuelle Comets

**Affiliations:** ^1^ INSERM, UMRS 1138, team 22, CRC, University Paris 5 University Paris 6 Paris France; ^2^ Federal Institute for Drugs and Medical Devices Bonn Germany; ^3^ INSERM, UMR 1123, Hôpital Robert‐Debré, APHP University Paris 7 Paris France; ^4^ Department of Medical Statistics University Medical Center Göttingen Göttingen Germany; ^5^ Statistics and Epidemiology, Division of Health Sciences, Warwick Medical School The University of Warwick Warwick UK; ^6^ INSERM, CIC 1414 University Rennes‐1 Rennes France; ^7^ INSERM, IAME UMR 1137 University Paris Diderot Paris France

**Keywords:** Dose‐finding, Dose‐toxicity relationship, Maximum tolerated dose, Pharmacokinetics, Phase I clinical trials

## Abstract

The aim of phase I clinical trials is to obtain reliable information on safety, tolerability, pharmacokinetics (PK), and mechanism of action of drugs with the objective of determining the maximum tolerated dose (MTD). In most phase I studies, dose‐finding and PK analysis are done separately and no attempt is made to combine them during dose allocation. In cases such as rare diseases, paediatrics, and studies in a biomarker‐defined subgroup of a defined population, the available population size will limit the number of possible clinical trials that can be conducted. Combining dose‐finding and PK analyses to allow better estimation of the dose‐toxicity curve should then be considered. In this work, we propose, study, and compare methods to incorporate PK measures in the dose allocation process during a phase I clinical trial. These methods do this in different ways, including using PK observations as a covariate, as the dependent variable or in a hierarchical model. We conducted a large simulation study that showed that adding PK measurements as a covariate only does not improve the efficiency of dose‐finding trials either in terms of the number of observed dose limiting toxicities or the probability of correct dose selection. However, incorporating PK measures does allow better estimation of the dose‐toxicity curve while maintaining the performance in terms of MTD selection compared to dose‐finding designs that do not incorporate PK information. In conclusion, using PK information in the dose allocation process enriches the knowledge of the dose‐toxicity relationship, facilitating better dose recommendation for subsequent trials.

## 1 Introduction

Dose‐finding studies are carried out in the very early stages of clinical evaluation, along with the first assessment of safety and pharmacokinetics (PK) in humans. The phase I dose‐finding study is usually the first trial in human subjects and has the goals of evaluating the safety of the tested drug, finding the maximum tolerated dose (MTD), and identifying a recommended dose (RD) for subsequent trials. The PK data collected during such a study provides a first step in describing the dose‐concentration and dose‐toxicity response relationships. Phase II studies then evaluate efficacy in patients, and may involve pharmacodynamic (PD) markers. Phase I trials usually include a small number of healthy volunteers (Chevret, 2006). An exception is in oncology, where, due to the potential high toxicity of drugs, even phase I trials are performed directly on patients (Chevret, 2006). In this case, PD measurements may be collected at this early stage in the drug development process (Lesko, 2007). PK/PD studies may be carried out both in the preclinical and in clinical stages, although informative PD biomarkers are generally available most frequently from phase II onwards (Derendorf et al., 2000). Since the effect of a drug usually depends on the drug–receptor interaction and on the responses of the physiological system, it is useful to monitor the concentration and take into account metabolic information in preclinical trials as well as in trials in humans. Moreover, regulatory agencies, such as the US Food and Drug Administration (FDA) and the European Medicines Agency (EMA), require both characterization of the concentration‐response and exposure‐toxicity relationships and dose justification for any new drug submission. Thus, modeling and simulation are increasingly used to streamline the drug development process (Mould and Upton, 2012). Moreover, after a drug candidate is selected for further development, detailed information on the metabolic processes and pharmacokinetics of the new drug is required by regulatory agencies (Jönsson et al., 2012).

Identifying the right dose or set of doses is a crucial objective in the drug development process: selecting too high a dose can result in an unacceptable toxicity profile, while selecting a dose that is too low increases the likelihood that the drug provides insufficient efficacy (Bretz et al., 2005). The dose escalation paradigm in phase I and/or phase II trials thus generally aims to avoid either unnecessary ineffective doses or toxic doses of an agent, that is to treat as many patients as possible within the therapeutic dose range. Due to the limited sample size, conventional statistical analysis could be inaccurate and adaptive designs have been proposed as they can potentially find the MTD sooner and limit the number of subjects used (Le Tourneau et al., 2009).

In the case of small population sizes, for example in rare diseases or pediatrics, it should be useful to take into account all the information collected during the trial, and to try to use PK data within the dose‐finding design. We expected that using all data available could be helpful in the recommendation of doses to be tested in subsequent trials, for example phase II and phase III. Standard dose‐finding designs like the 3+3 design or the CRM focus only on toxicity outcomes. This may be appropriate when simply targeting the highest dose subject to specific safety constraints but ignores the underlying dose‐toxicity relationship. PK information could help to give a better understanding of the entire dose‐toxicity relationship. In such situations, we propose to study and compare methods that use PK measurements in the dose allocation process in order to understand if and when this might be beneficial. In this paper, we will focus on small sample results, as dose‐finding phase I studies typically involve a limited number of subjects, but these methods could also be applied in larger studies.

The remainder of the paper is organized as follows. Section 2 describes and summarizes the motivation for our study. Section 3 introduces the statistical models and methods, either found in the literature and modified or newly proposed here. Section 4 outlines the simulation studies carried out and the results achieved. The last section includes discussion and recommendations.

## 2 Motivation

In phase I studies, even if dose‐finding and PK/PD analysis are carried out in the same trial, they are often conducted and reported independently in different sections in publications reporting trial results. A survey of the way pharmacokinetic analyses were reported in published phase I clinical trials in oncology was carried out in 2009 (Comets and Zohar, 2009): over 300 published papers were reviewed to evaluate how PK studies were presented and used in dose‐finding trials. In 84% of published papers, the PK analysis was clearly defined as a primary objective of the study at the same level as finding the MTD, but the dose‐finding study and the PK analysis were performed separately and described in distinct paragraphs. Only in 12% of the papers did the authors try, usually at the end of the trial, to retrospectively associate the observed toxicities or MTD with PK measurements. None of the clinical trials made any attempt to take into account the PK data during the trial in the dose‐allocation process. In some papers that did include a retrospective analysis linking PK data and dose‐finding there was a statistically significant relationship between toxicity and PK measures. For instance, in Broker et al. (2006), the authors found that patients for whom a DLT was observed, or there was a dose reduction after the first dose, had a mean AUC significantly different to patients not experiencing severe toxicity. Ajani et al. (2005) also found that patients who experienced a DLT had a statistically significantly higher AUC than patients who did not experience a DLT. By contrast, Fracasso et al. (2005) found that neither toxicity nor response was clearly related to the drug AUC or clearance. In these illustrations, the relation between DLT and PK was obtained retrospectively so it is unclear if the estimated MTD at the end of the trial would have been the same or different if the PK had also been used to guide the dose allocation process.

One possible hurdle to a more integrated approach is the difficulty of taking into account PK/PD measurements in the dose‐finding process. Dose‐finding methods using PK information include pharmacologically guided phase I trials (Collins et al., 1990), which use preclinical PK information to select the set of the doses tested in a dose‐escalation study, as well as extensions of the Continual Reassessment Method (CRM) (O'Quigley et al., 1990), such as the use of a parametric dose‐toxicity function that incorporates quantitative effects for both the dose of drug and a PK measure of exposure proposed in Piantadosi and Liu (1996). A middle ground was proposed by Patterson et al. (1999) and Whitehead et al. (2007), who proposed a Bayesian procedure with a nested hierarchical structure, in which PK/PD data are used to fit an overall dose–response relationship, so that PK/PD data are considered as dependent variables rather than as covariates. In both cases, decision theory is applied to maximize a chosen gain function in order to select the next dose. A special case is presented in O'Quigley et al. (2010), where, in the field of bridging studies, the dose associated with a mean PK response was selected based on linear regression analysis.

Our purpose in the present study is to determine what additional information on the MTD and the dose‐toxicity relationship is gained by incorporating PK information in the dose allocation process, and whether the benefit is sufficient to balance the added complexity of such a trial. The design and data collection will be more complex, as getting PK measures in real time during the trial can be challenging and expensive. PK data are generally collected and analyzed before going to Phase II, but usually not at the time of dose allocation in the ongoing Phase I study. On the other hand, in certain areas such as rare diseases, pediatrics, and biomarker‐specific subgroups, the size and nature of the population will impose logistic and ethical constraints on clinical trials, limiting the number of patients to be recruited as well as the amount of information that can be obtained for each subject. In such cases, during the early phase, instead of conducting the PK and the dose allocation separately, the possibility to combine both types of information to allow a better estimation of the dose should therefore be considered.

Our main motivation in this paper is therefore to propose, study, and compare methods that enable the use of PK measures in sequential Bayesian adaptive dose‐finding design, where Bayesian estimation takes into account prior information and the dose assigned to the next cohort is adapted sequentially using all information already accrued (sequential Bayesian adaptive designs). We considered different approaches developed for specific cases or diseases, and extended the underlying models to modify and adjust them to the same clinical setting. We then compared the methods, including also designs in which PK data are not used, through an extensive simulation study. We simulated subjects with PK and toxicity data based on a real example from a novel approach in oncology, inhibition of TGF‐β signaling to block tumor growth (Gueorguieva et al., 2014). The PK measure of exposure was the area under the concentration curve (AUC), and was considered to trigger toxicity when above a certain threshold. The AUC was included in the dose‐finding process in different ways, including being used as a covariate, as a dependent variable or in a hierarchical model. We evaluated the different approaches in terms of the probability of selecting the MTD correctly and the percentage of subjects underdosed or overdosed. We also investigated the ability of each method to estimate the dose‐toxicity curve, that is, to estimate correctly the probability of toxicity at each dose level tested in the trial. We propose to compare and discuss these methods in order to understand if there is a benefit from incorporating PK information and if so how this can best be achieved, and to give recommendations for practice.

## 3 Dose‐finding methods

In this section, we describe the methods selected to be compared in this work. Three Bayesian dose‐finding methods identified from the literature and modified to be applied in the same context are described in detail. In these methods PK measurements could enter in the model as covariates (Piantadosi and Liu, 1996; Whitehead et al., 2007) or as the dependent variable in regression (Patterson et al., 1999; Whitehead et al., 2007). Two variations of such methods are proposed and two other methods have been added to test the behaviors of the two philosophies “dose ‐ tox” and “dose ‐ auc ‐ tox.” The first approach, “dose ‐ tox,” is the most common and entails estimating the relationship between the probability of toxicity, $p_{T}$, and dose directly. Thus, this simply requires a statistical model to link $p_{T}$ and dose. The second approach, “dose ‐ auc ‐ tox,” instead, consists of estimating probabilities of toxicity of doses through the AUC, or some other PK measure of exposure. This involves use of two models relating AUC to dose and toxicity to AUC and then computation of $p_{T}$ as an expectation, that is, using notation described in detail below, $p_{T}\left(d\right)=\int_{x}P\left(Y=1|AUC=x\right)P\left(AUC=x|d\right)$. For each method, the dose‐toxicity model or the AUC‐toxicity model, the dose allocation rules and the method for recommendation of doses are described.

### Notation

Let $d_{k}$ be a dose in the set *D* of *K* possible doses $\left(d_{1},d_{2},\cdots ,d_{K}\right)$, $d_{\left[i\right]}\in D$ be the dose administered to the *i*‐th subject, $y_{i}$ be a binary variable that takes value 1 if the *i*‐th subject shows a DLT (dose‐limiting toxicity) and 0 otherwise, and $z_{i}$ be the logarithm of the AUC for the *i*‐th patient. Moreover, let $p_{T}$ denote the probability of toxicity, ***β*** the vector of the regression parameters (not in bold if there is only a single parameter), and *n* the sample size. Finally, let θ be the target probability. Other notation required for the description of each specific method are introduced.

### 3.1 PKCOV

The first method, which we will call PKCOV, is a modification of the method proposed by Piantadosi and Liu (1996) who suggested incorporating PK as covariate in a model for $p_{T}$ through the logit link.

**Dose‐toxicity model**: The PKCOV method dose‐toxicity model is

(1) $$\text{logit}\left(p_{T}\left(d_{k},\Delta {z_{d}}_{k},\beta \right)\right)=-\beta_{0}+\beta_{1}\log\left(d_{k}\right)+\beta_{2}\Delta {z_{d}}_{k}\;\forall d_{k}\in D,$$

where $\beta =(\beta_{1},\beta_{2})$, β_0_ is a constant and $\Delta {z_{d}}_{k}$ is the difference between the logarithm of population AUC at dose $d_{k}$ and *z*, the logarithm of AUC of the subject at the same dose. The original model uses $d_{k}$ instead of $\log(d_{k})$, but simulations from the PK model (described in the next section) showed that the relationship is linear with $\log(d_{k})$ and not $d_{k}$. Therefore, we also changed ΔAUC to Δz in order to have both the covariates on the same scale. The two covariates are not correlated if an underlying linear PK model is assumed, since Δz does not depend on dose but on PK parameters such as clearance. As in the original paper, we will use independent uniform priors for β_1_ and β_2_ so that the joint density function is given by $f\left(\beta_{1},\beta_{2}\right)={\left(u_{1}-l_{l}\right)}^{-1}{\left(u_{2}-l_{2}\right)}^{-1}$, where *u*_1_ and *l*_1_ are, respectively, the upper and the lower bound of the uniform distribution chosen for β_1_ so that $\beta_{1}∼U\left(l_{1},u_{1}\right)$, and similarly $\beta_{2}∼U\left(l_{2},u_{2}\right)$. The binomial likelihood, after *n* patients have been enrolled, is given by

(2) $$L_{n}\left(\beta |y\right)=\prod_{i=1}^{n}{\left[p_{T}\left(d_{\left[i\right]},\Delta {z_{d}}_{i},\beta \right)\right]}^{y_{i}}{\left[1-p_{T}\left(d_{\left[i\right]},\Delta {z_{d}}_{i},\beta \right)\right]}^{1-y_{i}}$$

with $p_{T}$ replaced by its explicit formula obtained from inverting Eq. (1).

**Dose allocation rules**: The dose chosen for the next cohort enrolled, after having collected data from the *i* previous patients, is the dose $d_{k}$ whose posterior probability of toxicity

(3) $$p_{T}\left(d_{k},{\hat{\beta }}_{1i}\right)=\frac{1}{1+e^{\beta_{0}-{\hat{\beta }}_{1i}\log\left(d_{k}\right)}}\;d_{k}\in D,$$

is nearest to the target probability, that is $d_{\left[i+1\right]}={\text{argmin}}_{d_{k}}\left|p_{T}\left(d_{k},{\hat{\beta }}_{1i}\right)-\theta \right|$. In Eq. (3) $\Delta {z_{d}}_{k}$ is replaced by zero, its expected value, as suggested in the original paper. In addition we added a no‐skipping dose rule, that is if not all doses have been tested previously, $d_{\left[i+1\right]}$ is chosen from $D^{*}\subset D$, a subset of *D* that contains all the doses already tested and the immediate next higher dose level.

**Recommended dose**: the final MTD estimate is given by $d_{\left[n+1\right]}$, that is the dose that would have been recommended for the (n+1)‐st subject enrolled in the trial.

### 3.2 PKLIM and PKCRM

The second method, which we will call PKLIM, is a modification of the method proposed by Patterson et al. (1999) and Whitehead et al. (2001), in which the authors used a hierarchical PK‐toxicity model in a cross‐over trial setting. Modifying their approach for a noncross‐over trial, that is removing random effects and using an underlying one compartment PK model, we obtain a Bayesian linear regression model.

**Dose‐AUC model**:

(4) $$z_{i}|\beta ,\nu ∼N\left(\beta_{0}+\beta_{1}\log d_{\left[i\right]},\nu^{2}\right)$$

where $\beta =(\beta_{0},\beta_{1})$ and $\beta |\nu ∼N_{2}\left(m,\nu^{2}G\right)$, ν∼Beta(a,b) are the prior distributions on (β|ν), the conditional mean, and ν, the standard deviation. *G* is a diagonal matrix and constants m, *G*, *a*, and *b* are chosen to represent prior knowledge. In contrast to the original model, we propose the use of a beta distribution for ν since this parameter is related to the interindividual variability of clearance, a PK parameter, and we assume that its value is between 0 and 1.

**Dose allocation rules**: the dose assigned to the next cohort, after having collected data from the *i* previous patients, is chosen so as to satisfy

(5) $$d_{\left[i+1\right]}={\text{argmin}}_{d_{k}}\left|P(z_{i+1}>\log\left(L\right)|\beta ={\hat{\beta }}_{i})-\theta \right|,$$

where *L* is a threshold set before starting the trial and ${\hat{\beta }}_{i}$ is the posterior mean of ***β***, computed after *i* patients. We changed the original dose allocation rule in which the dose assigned to the (i+1)‐st subject is chosen as the largest dose that satisfies the safety constraint $P(z_{i+1}>L|\beta ={\hat{\beta }}_{i})\leq \theta$, since simulations indicated that the original approach is less robust and, due to estimation problems, tends to be overly conservative. As with the PKCOV method, we added the no‐skipping dose rule.

**Recommended dose**: the MTD is defined as the dose recommended for the (n+1)‐st subject.

Since an assumption underlying the model is that DLTs are based on the AUC exceeding some threshold, the method could be applicable only when such a threshold is known. In order to try to avoid this problem, we propose a new method, PKCRM, which is the combination of PKLIM and the CRM using a power working model and normal prior on the parameter. In PKCRM the dose recommended for the next subject is the lowest of the doses recommended by the two methods. Thus the incorporation of the PKLIM method provides an additional safety check based on the AUC.

### 3.3 PKLOGIT

In the third method, called PKLOGIT, inspired by Whitehead et al. (2007) we use $z_{i}$ instead of dose $d_{\left[i\right]}$ as a covariate in a logistic regression model for $p_{T}$.

**AUC‐toxicity model**:

(6) $$\text{logit}\left(p_{T}\left(z,\beta \right)\right)=-\beta_{2}+\beta_{3}z,$$

with a bivariate uniform distribution as prior distribution for $\beta =(\beta_{2},\beta_{3})$, that is $f\left(\beta_{2},\beta_{3}\right)={\left(u_{2}-l_{2}\right)}^{-1}{\left(u_{3}-l_{3}\right)}^{-1}$ where *u*_2_ and *l*_2_ are, respectively, the upper and the lower bound of the uniform distribution chosen for β_2_, $U(l_{2},u_{2})$, and *u*_3_ and *l*_3_ for β_3_, $U(l_{3},u_{3})$. These priors, although not as used by Whitehead et al. (2007), have been chosen in order to make this method more comparable with other methods presented in this paper. Lastly, we borrowed the hierarchical model of PKLIM for $z_{i}$ that is necessary for computing the posterior probability of toxicity associated with each dose. The likelihood and the posterior means for parameters are obtained by substituting the explicit form of $p_{T}\left(z,\beta \right)$, obtained by inverting Eq. (6), and the actual prior distribution into Eq. (2).

**Dose allocation rules**: Since we did not include the PD variable in our work, we modified the dose allocation rules shown in the original paper (Whitehead et al., 2007) and the dose given to the next cohort, after having enrolled *i* patients, is the one whose predictive probability

(7) $$P\left(y_{i+1}=1|\beta ={\hat{\beta }}_{i}\right)=E\left[\frac{1}{1+e^{{\hat{\beta }}_{2i}-{\hat{\beta }}_{3i}z_{i+1}}}\right]=\int \frac{1}{1+e^{{\hat{\beta }}_{2i}-{\hat{\beta }}_{3i}z_{i+1}}}g\left(z_{i+1}\right)\;dz_{i+1},$$

is the nearest to the target probability θ. In Eq. (10), *g* is the predictive normal density of $z_{i+1}$ given ${\hat{\beta }}_{i}$ where ${\hat{\beta }}_{i}={\left({\hat{\beta }}_{0},{\hat{\beta }}_{1},{\hat{\beta }}_{2},{\hat{\beta }}_{3}\right)}_{i}$ have been estimated across all doses. As in the previous models, we added the no‐skipping dose rule.

**Recommended dose**: the MTD is defined as the dose $d_{\left[n+1\right]}\in D$ recommended for the (n+1) subject.

### 3.4 PKPOP

**Dose‐toxicity model**: A variation of PKLOGIT, that we call PKPOP, arises by replacing *z* with $z_{k,pop}$ in Eq. (6), where $z_{k,pop}$ is the mean value of the logarithm of AUC at dose $d_{k}$ predicted by the hierarchical model in PKLIM. In other words, we replace the observed value for the patient with the population mean value. In this way, we pass directly to the population level.

**Dose allocation rules**: the dose for the next cohort is the dose $d_{k}$ whose posterior probability of toxicity

(8) $$p_{T}\left(z_{k,pop}|{\hat{\beta }}_{i},d_{k}\right)=\frac{1}{1+e^{{\hat{\beta }}_{3i}-{\hat{\beta }}_{4i}z_{k,pop}}}\;d_{k}\in D,$$

is nearest to the target probability, that is $d_{\left[i+1\right]}={\text{argmin}}_{d_{k}}\left|p_{T}\left(z_{k,pop}|{\hat{\beta }}_{i},d_{k}\right)-\theta \right|$.

**Recommended dose**: As for other methods the MTD is defined as the dose $d_{\left[n+1\right]}\in D$ recommended for the (n+1)‐st subject.

### 3.5 PKTOX

The fourth method, called PKTOX, that estimates probabilities of toxicity of doses passing through the AUC, is essentially the PKLOGIT method with a probit regression model replacing the logistic regression.

**AUC‐toxicity model**:

(9) $$p_{T}\left(z,\beta \right)=\Phi \left(-\beta_{2}+\beta_{3}z\right),$$

with Φ represents the standard cumulative normal distribution, replaces Eq. (6) in PKLOGIT method. The use of a probit instead of a logistic regression model is consistent with the example chosen, described in the next section, and is studied to verify what happens in the best case, that is when the statistical analysis and simulation models coincide.

**Dose allocation rules**: As in PKLOGIT, the dose given to the next cohort is the one with predictive probability

(10) $$P\left(y_{i+1}=1|\beta ={\hat{\beta }}_{i}\right)=E\left[\Phi \left(-\beta_{2}+\beta_{3}z_{i+1}\right)\right]=\int \Phi \left(-\beta_{2}+\beta_{3}z_{i+1}\right)g\left(z_{i+1}\right)\;dz_{i+1},$$

nearest to the target probability θ. As in the previous models, a no‐skipping dose rule is added.

**Recommended dose**: the MTD is defined as the dose $d_{\left[n+1\right]}\in D$ recommended for the (n+1)‐st subject.

### 3.6 DTOX

The last method, that we will call DTOX, follows the usual way of estimating $p_{T}$ versus dose directly without the PK measurements and is included to check the behavior of this standard method.

**Dose‐toxicity model**: the dose‐toxicity model is

(11) $$p_{T}\left(d_{k},\beta \right)=\Phi \left(-\beta_{0}+\beta_{1}\log\left(d_{k}\right)\right)\;\forall d_{k}\in D,$$

with a bivariate uniform distribution chosen as the prior distribution for $\beta =(\beta_{0},\beta_{1})$, that is $f\left(\beta_{0},\beta_{1}\right)={\left(u_{0}-l_{0}\right)}^{-1}{\left(u_{1}-l_{1}\right)}^{-1}$. The likelihood can be computed substituting Eq. (11) into Eq. (2).

**Dose allocation rules**: the dose chosen for the next cohort is the dose $d_{k}$ whose posterior probability of toxicity $p_{T}\left(d_{k},{\hat{\beta }}_{i}\right)=\Phi \left(-{\hat{\beta }}_{0}+{\hat{\beta }}_{1}\log\left(d_{k}\right)\right),d_{k}\in D$, is nearest to the target probability θ.

**Recommended dose**: As for all other methods, the MTD is defined as the dose $d_{\left[n+1\right]}\in D$ recommended for the (n+1)‐st subject.

## 4 Simulation study

### 4.1 Simulation settings

In the context proposed for our simulations, toxicity is related to a PK measure of exposure, more precisely to the AUC, the area under the curve in a plot of concentration of the drug in blood plasma against time. We could not follow the usual way of simulating toxicity in dose‐finding simulation trials, in which a set of probabilities, one for each dose, is set and patient's responses are drawn from Bernoulli distributions. Rather, we first simulate PK data and then simulate toxicity based on the AUC values. In order to achieve this we started with the example based on the PK model for the TGF‐β inhibitor LY2157299 in patients with glioma (Gueorguieva et al., 2014; Bueno et al., 2008). TGF‐β signaling is an important growth regulator in advanced cancer and a novel treatment approach consists of inhibiting TGF‐β signaling in order to simultaneously inhibit tumor spread and neo‐angiogenesis and improve the patient's anti‐tumor immune response.

We used a simplified PK model as in Lestini et al. (2015), that is a first‐order absorption linear one compartment model, parametrized by $k_{a}$, the absorption rate constant for oral administration, CL, the clearance of elimination, and *V*, the volume of distribution. The equation that expresses the concentration c(t) at time *t* after the drug administration $d_{k}$ at time 0 is then

(12) $$c\left(t\right)=\frac{d_{k}}{V}\frac{k_{a}}{k_{a}-CL/V}\left(e^{-(CL/V)t}-e^{-k_{a}t}\right).$$

**Figure 1:**
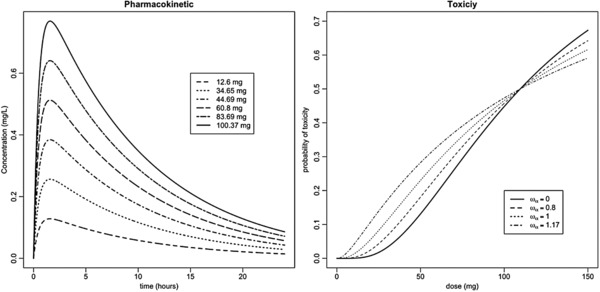
Left: plot of the concentration of the drug versus time for 12.6, 34.65, 44.69, 60.8, 83.69, and 100.37 mg with $k_{a}=2$ h^−1^, CL=10 L h^−1^ , and V=100 L. Right: probability of toxicity versus dose (Eq. (13)) for several values of $\omega_{\alpha }$ with $\tau_{T}=10.96$ mg L^−1^ h, ω CL =0.7, and CL=10 L h^−1^.

Figure 1 left hand plot shows the behavior of the model described above, with $k_{a}=2$ h^−1^, CL=10 L h^−1^ , and V=100 L for the doses of 12.6, 34.65, 44.69, 60.8, 83.69, and 100.37 mg.

With the exception of $k_{a}$ that is taken to be fixed with $k_{a}=2$ h^−1^, we suppose that subjects' PK parameters come from log‐normal distributions with means equal to the values proposed in Lestini et al. (2015); CL=10 L h^−1^ and V=100 L, and standard deviations $\omega_{CL}=\omega_{V}$ equal to 70% or 30% in the different scenarios. Based on this, we simulated several scenarios, with PK data simulated from this model with different probabilities of toxicity.

In order to relate PK and toxicity, we introduced a measure of subject sensitivity in our model. We set a linear function of AUC, that is s(AUC)=αAUC, in which α is a sensitivity parameter of the patient assumed to come from a log‐normal distribution with mean equals to 1 and standard deviation $\omega_{\alpha }$. We then fixed a threshold, $\tau_{T}$, and assumed that a subject would experience a DLT at a given dose if *s*(AUC) is equal to or greater than $\tau_{T}$. Therefore, since AUC is computed as $d_{k}/CL$, we have that

(13) $$p_{T}\left(d_{k}\right)=\Phi \left(\frac{\log d_{k}-\log\tau_{T}-\log CL}{\sqrt{\omega_{CL}^{2}+\omega_{\alpha }^{2}}}\right),$$

where Φ() represents the cumulative function of a standard normal distribution.

Changing the values of $\omega_{\alpha }$ leads to different scenarios for $p_{T}$, as shown in Fig. 1 (right hand plot). Seven different scenarios were simulated. Values of τ and α were chosen in order to have the MTD at a specific position. Table 1 shows the parameters used in the different scenarios. The first scenario is the closest to the models presented in Gueorguieva et al. (2014) and Lestini et al. (2015) and the doses used in the simulated trial were chosen to achieve the desired probabilities of toxicity, that is (0.001, 0.05, 0.1, 0.2, 0.35, 0.45), under this scenario by inverting Eq. (13) with $\omega_{\alpha }$ set to zero so that the threshold is the same for all patients. We then modified the threshold in order to simulate trials in which the MTD was in another position without changing the way in which toxicity was simulated (scenarios 2 and 3). In the next two scenarios (4 and 5), we added variability in the threshold to explore situations in which patients will experience toxicity according to a subject‐specific threshold, which is likely to be more representative of actual toxicity occurrence. Finally, we analyzed scenarios where the IIV is decreased from 0.7 to 0.3 (scenarios 6 and 7) to evaluate a situation where we expect the impact of taking PK information into account to increase.

**Table 1:** Parameters of simulated scenarios

Scenario	$k_{a}$ (h^−1^)	CL (L h^−1^)	*V* (L)	$\omega_{CL}=\omega_{V}$	$\omega_{\alpha }$	$\tau_{T}$ (mg L^−1^ h)
Scenario 1	2	10	100	0.7	0	10.96
Scenario 2	2	10	100	0.7	0	15.09
Scenario 3	2	10	100	0.7	0	18.10
Scenario 4	2	10	100	0.7	1.17	10.96
Scenario 5	2	10	100	0.7	0.8	10.96
Scenario 6	2	10	100	0.3	0	10.96
Scenario 7	2	10	100	0.3	1	10.96

Notes. Each line describes one scenario and values of $k_{a}$, CL, *V*, $\omega_{CL}$, $\omega_{V}$, $\omega_{\alpha ,}$ and $\tau_{T}$ are shown.

In our setting, we took the measure of exposure to be area under the concentration time curve (AUC), but, depending on the mechanism of action of the drug, $C_{max}$ or some other measurements of exposure could be used instead.

### Trial settings

In order to compare the dose‐finding methods, we simulated subject responses to all doses for each trial. We started by drawing individual PK parameters from the population distributions for each subject, as described in the previous subsection. Then, for each dose level and for all patients we computed concentration measurements to which we added a proportional error drawn from a normal distribution with zero mean and standard deviation of 20%. Finally, we simulated toxicity values at each dose level using Eq. (13). Each simulated dataset was stored and, for each dose‐finding method, subject responses were read from this complete dataset. In this way, when two methods coincide in dose allocation, the results of the simulated runs are the same. For each scenario, 1000 trials were simulated.

For the design, we supposed a cohort size of 1 subject and a fixed sample size of 30. We escalated the dose one by one until the first toxicity and then we switched to the chosen model design (two‐stage design). If any toxicity is seen in the trial, estimation methods are applied at the end of the trial. In order to estimate the AUC, we assumed a one‐compartmental model with linear elimination and we estimated parameters for each patient through nonlinear least squares (NLS) method. Ten samples from simulated concentrations were used in NLS. For the PKCOV method, the population AUC at dose $d_{k}$ was computed as the mean of the AUCs of the previous subjects allocated at dose $d_{k}$. As in Gueorguieva et al. (2014), we set θ equal to 0.2, that is the MTD is the dose for which 20% of subjects exhibit DLT. We applied PKCRM with several *L* instead of PKLIM alone, since PKLIM depends only on *L* and gives the same result in all scenarios. We set *L* equal to 7.05, 10.96, 15.09, and 18.1 mg L^−1^ h. The last three represent real thresholds $\tau_{T}$ used to simulate scenarios, to put ourselves in the best case possible for the PKCRM in at least one scenario. The skeleton of CRM for all the trials was set as p=(0.01,0.05,0.1,0.2,0.35,0.45), that is the real probabilities of toxicity related to the first scenario except for the first dose, and priors and other constants are summarized in Table 2. Priors were chosen for the case in which we have good previous information for scenario 1. This is the case in which, for example, we have information from preclinical data. When this was impossible, for example for PKLOGIT and PKTOX (since it is difficult to approximate a step toxicity function through a logistic or Φ function), we selected values which, after preliminary simulations, gave us acceptable results for several settings, as is usually done in the absence of prior information.

**Table 2:** Dose‐finding models parameters and priors; $ir_{1}=-14.76$ and $sr_{1}=3.23$ are, respectively, the intercept and the slope obtained by a logistic regression with real probability of scenario 1 at doses in *D* versus logarithm of doses; $CL_{pop}=10$ is population clearance; $ir_{2}=6.71$ and $sr_{2}=1.43$ represents the intercept and the slope value of formula (11) when parameters of scenario 1 are used

Name	Model	Likelihood	Priors and constants	Posterior $p_{T}$
PKCOV	$\text{logit}\left(p_{T}\left(d_{k},\Delta {z_{d}}_{k},\beta \right)\right)=-\beta_{0}+\beta_{1}\log\left(d_{k}\right)+\beta_{2}\Delta {z_{d}}_{k}$	$\prod_{j=1}^{n}{\left[p_{T}\left(d_{j},\beta \right)\right]}^{y_{j}}{\left[1-p_{T}\left(d_{j},\beta \right)\right]}^{1-y_{j}}$	$\beta_{0}=-ir_{1}$	$\frac{1}{1+e^{\beta_{0}-{\hat{\beta }}_{1i}\log\left(d_{k}\right)}}$
			$\beta_{1}∼U\left(l_{1},u_{1}\right)$	
			$\beta_{2}∼U\left(0,5\right)$	
			$l_{1}=\max\left(0,sr_{1}-5\right)$	
			$u_{1}=sr_{1}+5$	
PKLIM	$z_{i}|\beta ,\nu ∼N\left(\beta_{0}+\beta_{1}\log d_{i},\nu^{2}\right)$	${\left(\frac{1}{2\pi \nu^{2}}\right)}^{n/2}\exp\left(-\frac{\sum_{j=1}^{n}{\left(z_{j}-\beta_{0}-\beta_{1}\log d_{j}\right)}^{2}}{2\nu^{2}}\right)$	$\beta |\nu ∼N_{2}\left(m,\nu^{2}G\right)$	$P(z_{i+1}>L|\beta ={\hat{\beta }}_{i})$
			ν∼Beta(1,1)	
			$m=(-\log CL_{pop},1)$	
			G=diag(1000,1000)	
PKCRM	$z_{i}|\beta ,\nu ∼N\left(\beta_{0}+\beta_{1}\log d_{i},\nu^{2}\right)$ + $p_{T}\left({\tilde{d}}_{k},\beta \right)={\tilde{d}}_{k}^{\beta }$ (CRM)	${\left(\frac{1}{2\pi \nu^{2}}\right)}^{n/2}\exp\left(-\frac{\sum_{j=1}^{n}{\left(z_{j}-\beta_{0}-\beta_{1}\log d_{j}\right)}^{2}}{2\nu^{2}}\right)$ + $\prod_{j=1}^{n}{\left[p_{T}\left(d_{j},\beta \right)\right]}^{y_{j}}{\left[1-p_{T}\left(d_{j},\beta \right)\right]}^{1-y_{j}}$	PKLIM priors	$P(z_{i+1}>L|\beta ={\hat{\beta }}_{i})$ and ${\tilde{d}}_{k}^{{\hat{\beta }}_{i}}$
			Skeleton CRM =	
			(0.01,0.05,0.1,0.2,0.35,0.45)	
			β∼N(0,1.34)	
PKLOGIT	$z_{i}|\beta ,\nu ∼N\left(\beta_{0}+\beta_{1}\log d_{i},\nu^{2}\right)$ + $\text{logit}\left(p_{T}\left(z,\beta \right)\right)=-\beta_{2}+\beta_{3}z$	${\left(\frac{1}{2\pi \nu^{2}}\right)}^{n/2}\exp\left(-\frac{\sum_{j=1}^{n}{\left(z_{j}-\beta_{0}-\beta_{1}\log d_{j}\right)}^{2}}{2\nu^{2}}\right)$ + $\prod_{j=1}^{n}{\left[p_{T}\left(z_{j},\beta \right)\right]}^{y_{j}}{\left[1-p_{T}\left(z_{j},\beta \right)\right]}^{1-y_{j}}$	PKLIM priors	$\int \frac{1}{1+e^{{\hat{\beta }}_{2i}-{\hat{\beta }}_{3i}z_{i+1}}}g\left(z_{i+1}\right)\;dz_{i+1}$
			$\beta_{2}∼U\left(0,20\right)$	
			$\beta_{3}∼U\left(0,10\right)$	
PKPOP	$z_{i}|\beta ,\nu ∼N\left(\beta_{0}+\beta_{1}\log d_{i},\nu^{2}\right)$ + $\text{logit}\left(p_{T}\left(z_{k,pop},\beta \right)\right)=-\beta_{3}+\beta_{4}z_{k,pop}$	${\left(\frac{1}{2\pi \nu^{2}}\right)}^{n/2}\exp\left(-\frac{\sum_{j=1}^{n}{\left(z_{j}-\beta_{0}-\beta_{1}\log d_{j}\right)}^{2}}{2\nu^{2}}\right)$ + $\prod_{j=1}^{n}{\left[p_{T}\left(z_{j},\beta \right)\right]}^{y_{j}}{\left[1-p_{T}\left(z_{j},\beta \right)\right]}^{1-y_{j}}$	PKLIM priors	$\frac{1}{1+e^{{\hat{\beta }}_{3i}-{\hat{\beta }}_{4i}z_{k,pop}}}$
			$\beta_{3}∼U\left(0,10\right)$	
			$\beta_{4}∼U\left(0,5\right)$	
PKTOX	$z_{i}|\beta ,\nu ∼N\left(\beta_{0}+\beta_{1}\log d_{i},\nu^{2}\right)$ + $p_{T}\left(z,\beta \right)=\Phi \left(-\beta_{2}+\beta_{3}z\right)$	${\left(\frac{1}{2\pi \nu^{2}}\right)}^{n/2}\exp\left(-\frac{\sum_{j=1}^{n}{\left(z_{j}-\beta_{0}-\beta_{1}\log d_{j}\right)}^{2}}{2\nu^{2}}\right)$ + $\prod_{j=1}^{n}{\left[p_{T}\left(z_{j},\beta \right)\right]}^{y_{j}}{\left[1-p_{T}\left(z_{j},\beta \right)\right]}^{1-y_{j}}$	PKLIM priors	$\int \Phi \left(-{\hat{\beta }}_{2i}+{\hat{\beta }}_{3i}z_{i+1}\right)g\left(z_{i+1}\right)\;dz_{i+1}$
			$\beta_{2}∼U\left(0,20\right)$	
			$\beta_{3}∼U\left(0,10\right)$	
DTOX	$p_{T}\left(d_{k},\beta \right)=\Phi \left(-\beta_{0}+\beta_{1}\log\left(d_{k}\right)\right)$	$\prod_{j=1}^{n}{\left[p_{T}\left(z_{j},\beta \right)\right]}^{y_{j}}{\left[1-p_{T}\left(z_{j},\beta \right)\right]}^{1-y_{j}}$	$\beta_{q}∼U\left(l_{q},u_{q}\right)\;q=0,1$	$\Phi (-{\hat{\beta }}_{0i}+{\hat{\beta }}_{1i}d_{k})$
			$l_{0}=\max\left(0,ir_{2}-10\right)$	
			$u_{0}=ir_{2}+10$	
			$l_{1}=\max\left(0,sr_{2}-5\right)$	
			$u_{1}=sr_{1}+5$	

### 4.2 Results

**Figure 2:**
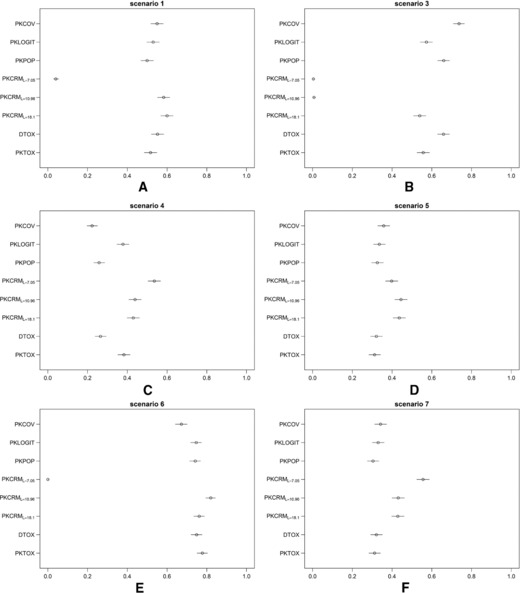
PCS for all the methods through seven scenarios. The estimated probability of toxicity with Clopped‐Pearson 95% confidential intervals after 1000 simulations is plotted for each method. Scenario 2 is omitted since it was intermediate between scenarios 1 and 3, as well as the PKCRM${}_{L=15.09}$ method, which behaved as expected in between PKCRM${}_{L=10.96}$ and PKCRM${}_{L=18.1}$.

In Fig. 2, we summarize the results on the probability of correct selection (PCS), that is, the percentage of simulated trials in which the correct MTD recommendation was made. We plotted the estimated probability of toxicity with Clopper‐Pearson 95% credible intervals after 1000 simulations for each method. To improve the layout of the figure, we decided to show only six scenarios, omitting Scenario 2 that was intermediate between scenarios 1 and 3, as well as the PKCRM${}_{L=15.09}$ method, which behaved as expected in between PKCRM${}_{L=10.96}$ and PKCRM${}_{L=18.1}$. Tables 3, 4, and 5 give details of PCS, and the probabilities of overdosing and under‐dosing for all methods in the 7 scenarios. Results are divided in three tables, according to characteristics of the scenarios. In Table 3 results from scenarios with $\omega_{CL}=\omega_{V}=0.7$ and without variability in α are summarized, Table 4 shows results for scenarios with $\omega_{CL}=\omega_{V}=0.7$, fixed threshold $\tau_{T}=10.96$ and several $\omega_{\alpha }$, and Table 5 shows results of scenarios with less IIV, $\omega_{CL}=\omega_{V}=0.3$, and $\tau_{T}=10.96$.

**Table 3:** Percentage of dose selection at the end of the trials, percentage of dose allocation and median, minimum, and maximum number of DLT for scenarios with $\omega_{CL}=\omega_{V}=0.7$, $\omega_{\alpha }=0$, and several $\tau_{T}$

Method	% dose selection						% dose allocation						number of DLTs		
	1	2	3	4	5	6	1	2	3	4	5	6	median (n)	min‐max	
Scenario 1															
$\tau_{T}=10.96$, MTD = dose level 4	*0.001*	*0.05*	*0.1*	*0.2*	*0.35*	*0.45*									
PKCOV	0.054	0.015	0.177	**0.550**	0.163	0.041	0.087	0.067	0.188	0.370	0.172	0.116	6	1	11
PKLOGIT	0.066	0.032	0.276	**0.530**	0.088	0.008	0.117	0.105	0.251	0.350	0.112	0.065	5	1	10
PKPOP	0.051	0.030	0.199	**0.500**	0.202	0.018	0.082	0.080	0.200	0.345	0.194	0.099	6	1	12
PKCRM${}_{L=7.05}$	0.104	0.381	0.475	**0.040**	0	0	0.137	0.353	0.363	0.086	0.025	0.036	3	1	9
PKCRM${}_{L=10.96}$	0.055	0.017	0.259	**0.583**	0.083	0.003	0.087	0.083	0.265	0.407	0.111	0.046	5	1	11
PKCRM${}_{L=15.09}$	0.030	0.013	0.202	**0.591**	0.157	0.007	0.067	0.075	0.216	0.409	0.170	0.062	6	1	11
PKCRM${}_{L=18.1}$	0.020	0.014	0.196	**0.600**	0.161	0.009	0.058	0.076	0.215	0.410	0.176	0.065	6	1	11
DTOX	0.055	0.016	0.195	**0.552**	0.168	0.014	0.087	0.071	0.211	0.369	0.176	0.085	6	1	11
PKTOX	0.069	0.038	0.279	**0.517**	0.090	0.007	0.117	0.109	0.247	0.353	0.112	0.062	5	1	10
Scenario 2															
$\tau_{T}=15.09$, MTD = dose level 5	*0*	*0.012*	*0.041*	*0.097*	*0.2*	*0.28*									
PKCOV	0.020	0	0.014	0.203	**0.353**	0.410	0.052	0.041	0.068	0.198	0.237	0.404	6	1	10
PKLOGIT	0.036	0.004	0.030	0.307	**0.412**	0.211	0.072	0.052	0.095	0.261	0.268	0.251	5	1	10
PKPOP	0.019	0.009	0.046	0.195	**0.428**	0.303	0.052	0.049	0.091	0.189	0.285	0.335	5	1	10
PKCRM${}_{L=7.05}$	0.063	0.401	0.491	0.043	**0.001**	0.001	0.098	0.352	0.356	0.079	0.031	0.084	2	1	9
PKCRM${}_{L=10.96}$	0.029	0.009	0.167	0.640	**0.148**	0.007	0.061	0.060	0.199	0.438	0.146	0.097	3	1	9
PKCRM${}_{L=15.09}$	0.021	0	0.016	0.299	**0.484**	0.180	0.054	0.043	0.084	0.284	0.330	0.206	5	1	10
PKCRM${}_{L=18.1}$	0.017	0	0.005	0.250	**0.499**	0.229	0.050	0.042	0.072	0.252	0.341	0.243	5	1	10
DTOX	0.020	0.001	0.018	0.219	**0.450**	0.292	0.053	0.042	0.081	0.216	0.297	0.311	5	1	10
PKTOX	0.036	0.004	0.034	0.320	**0.402**	0.204	0.072	0.052	0.094	0.264	0.273	0.244	5	1	10
Scenario 3															
$\tau_{T}=18.1$, MTD = dose level 6	*0*	*0.009*	*0.023*	*0.06*	*0.135*	*0.2*									
PKCOV	0.013	0	0.005	0.070	0.175	**0.737**	0.045	0.036	0.049	0.111	0.159	0.600	5	0	11
PKLOGIT	0.022	0.001	0.006	0.098	0.300	**0.573**	0.057	0.042	0.063	0.143	0.223	0.472	4	0	9
PKPOP	0.012	0.002	0.017	0.072	0.237	**0.660**	0.045	0.039	0.061	0.117	0.199	0.539	4	0	9
PKCRM${}_{L=7.05}$	0.046	0.417	0.493	0.039	0.001	**0.004**	0.082	0.342	0.341	0.071	0.032	0.132	1	0	8
PKCRM${}_{L=10.96}$	0.019	0.008	0.160	0.645	0.161	**0.007**	0.052	0.056	0.185	0.427	0.139	0.141	2	0	7
PKCRM${}_{L=15.09}$	0.014	0	0.010	0.203	0.459	**0.314**	0.047	0.039	0.067	0.222	0.324	0.302	4	0	9
PKCRM${}_{L=18.1}$	0.011	0	0	0.078	0.372	**0.539**	0.045	0.037	0.053	0.153	0.298	0.415	4	0	9
DTOX	0.013	0.000	0.005	0.066	0.257	**0.659**	0.045	0.037	0.054	0.121	0.215	0.528	4	0	9
PKTOX	0.022	0.001	0.008	0.105	0.307	**0.557**	0.058	0.043	0.062	0.146	0.229	0.463	4	0	9

Notes. Real percentage of toxicity of each dose is written in italics.

**Table 4:** Percentage of dose selection at the end of the trials, percentage of dose allocation and median, minimum, and maximum number of DLT for scenarios with interindividual variability of 70%, $\tau_{T}=10.96$, and several $\omega_{\alpha }$

Method	% dose selection						% dose allocation						number of DLTs		
	1	2	3	4	5	6	1	2	3	4	5	6	median (n)	min‐max	
Scenario 4															
$\omega_{\alpha }=1.17$, MTD = dose level 2	*0.056*	*0.199*	*0.255*	*0.333*	*0.422*	*0.474*									
PKCOV	0.315	**0.223**	0.299	0.134	0.024	0.005	0.309	0.203	0.234	0.157	0.057	0.040	7	1	14
PKLOGIT	0.290	**0.378**	0.233	0.074	0.015	0.010	0.335	0.282	0.186	0.113	0.042	0.042	6	1	11
PKPOP	0.254	**0.258**	0.297	0.147	0.038	0.006	0.264	0.222	0.237	0.168	0.068	0.041	7	1	13
PKCRM${}_{L=7.05}$	0.211	**0.536**	0.246	0.007	0	0	0.258	0.428	0.232	0.052	0.014	0.016	6	1	11
PKCRM${}_{L=10.96}$	0.121	**0.439**	0.324	0.112	0.004	0	0.185	0.336	0.274	0.155	0.033	0.017	7	1	12
PKCRM${}_{L=15.09}$	0.104	**0.433**	0.332	0.113	0.017	0.001	0.169	0.332	0.266	0.160	0.049	0.023	7	1	13
PKCRM${}_{L=18.1}$	0.099	**0.430**	0.337	0.115	0.016	0.003	0.166	0.333	0.266	0.160	0.051	0.024	7	1	13
DTOX	0.287	**0.265**	0.288	0.130	0.025	0.005	0.292	0.221	0.238	0.160	0.057	0.032	7	1	13
PKTOX	0.307	**0.383**	0.212	0.075	0.015	0.008	0.348	0.286	0.182	0.106	0.039	0.038	6	1	12
Scenario 5															
$\omega_{\alpha }=0.8$, MTD = dose level 3	*0.021*	*0.139*	*0.199*	*0.29*	*0.4*	*0.467*									
PKCOV	0.190	0.161	**0.358**	0.243	0.040	0.008	0.207	0.166	0.266	0.226	0.081	0.054	7	1	13
PKLOGIT	0.169	0.283	**0.336**	0.172	0.029	0.011	0.235	0.257	0.239	0.165	0.056	0.048	6	1	12
PKPOP	0.207	0.221	**0.326**	0.191	0.050	0.005	0.226	0.205	0.256	0.193	0.076	0.044	7	1	13
PKCRM${}_{L=7.05}$	0.132	0.454	**0.398**	0.016	0	0	0.185	0.406	0.303	0.065	0.019	0.021	5	1	10
PKCRM${}_{L=10.96}$	0.058	0.237	**0.445**	0.245	0.015	0	0.118	0.243	0.331	0.237	0.047	0.024	6	1	12
PKCRM${}_{L=15.09}$	0.034	0.238	**0.436**	0.257	0.032	0.003	0.100	0.238	0.314	0.246	0.070	0.032	6	1	12
PKCRM${}_{L=18.1}$	0.031	0.239	**0.436**	0.262	0.029	0.003	0.096	0.239	0.313	0.245	0.072	0.034	6	1	12
DTOX	0.232	0.231	**0.321**	0.178	0.035	0.003	0.249	0.204	0.261	0.186	0.064	0.037	7	1	13
PKTOX	0.170	0.317	**0.312**	0.160	0.030	0.011	0.242	0.264	0.234	0.160	0.055	0.044	6	1	12

Notes. Real percentage of toxicity of each dose is written in italics.

**Table 5:** Percentage of dose selection at the end of the trials, percentage of dose allocation and median, minimum, and maximum number of DLT for scenarios with interindividual variability of 30%, $\tau_{T}=10.96$, and several $\omega_{\alpha }$

Method	% dose selection						% dose allocation						number of DLTs		
	1	2	3	4	5	6	1	2	3	4	5	6	median (n)	min‐max	
Scenario 6															
$\omega_{\alpha }=0$, MTD = dose level 5	*0*	*0*	*0.001*	*0.025*	*0.184*	*0.385*									
PKCOV	0	0	0	0.080	**0.672**	0.248	0.033	0.033	0.038	0.164	0.424	0.308	6	2	10
PKLOGIT	0	0	0	0.143	**0.747**	0.110	0.034	0.036	0.055	0.240	0.443	0.193	5	1	10
PKPOP	0	0	0	0.113	**0.742**	0.145	0.033	0.034	0.038	0.165	0.469	0.262	6	2	10
PKCRM${}_{L=7.05}$	0	0.001	0.518	0.481	**0**	0	0.033	0.056	0.486	0.321	0.033	0.071	1	1	4
PKCRM${}_{L=10.96}$	0	0	0	0.129	**0.820**	0.051	0.033	0.033	0.041	0.255	0.517	0.120	5	1	8
PKCRM${}_{L=15.09}$	0	0	0	0.093	**0.763**	0.144	0.033	0.033	0.038	0.183	0.492	0.219	5	2	9
PKCRM${}_{L=18.1}$	0	0	0	0.093	**0.762**	0.145	0.033	0.033	0.038	0.183	0.492	0.220	5	2	9
DTOX	0	0	0	0.102	**0.749**	0.149	0.033	0.033	0.038	0.179	0.470	0.246	6	2	9
PKTOX	0.001	0	0	0.118	**0.778**	0.103	0.034	0.036	0.055	0.240	0.466	0.170	5	1	10
Scenario 7															
$\omega_{\alpha }=1$, MTD = dose level 3	*0.019*	*0.135*	*0.195*	*0.286*	*0.398*	*0.466*									
PKCOV	0.193	0.144	**0.322**	0.287	0.048	0.006	0.218	0.163	0.242	0.239	0.085	0.053	6.5	1	13
PKLOGIT	0.197	0.212	**0.330**	0.208	0.040	0.013	0.242	0.213	0.250	0.185	0.067	0.043	6	1	12
PKPOP	0.184	0.153	**0.304**	0.275	0.079	0.005	0.203	0.170	0.243	0.232	0.102	0.051	7	1	13
PKCRM${}_{L=7.05}$	0.015	0.257	**0.556**	0.172	0	0	0.087	0.272	0.453	0.151	0.016	0.021	6	1	10
PKCRM${}_{L=10.96}$	0.012	0.248	**0.431**	0.275	0.034	0.000	0.082	0.252	0.313	0.261	0.069	0.024	6	3	12
PKCRM${}_{L=15.09}$	0.012	0.243	**0.428**	0.275	0.040	0.002	0.082	0.251	0.308	0.250	0.077	0.032	6	3	12
PKCRM${}_{L=18.1}$	0.015	0.240	**0.429**	0.275	0.038	0.003	0.084	0.249	0.308	0.248	0.077	0.033	6	1	12
DTOX	0.191	0.174	**0.321**	0.256	0.054	0.004	0.216	0.181	0.245	0.229	0.085	0.044	6	1	13
PKTOX	0.219	0.236	**0.312**	0.185	0.043	0.005	0.258	0.226	0.248	0.171	0.060	0.037	6	1	12

Notes. Real percentage of toxicity of each dose is written in italics.

In the two upper plots of Fig. 2 scenarios 1 and 3 are shown: they correspond to simulations with the same PK parameter values, without variability in α; the only difference is in the threshold $\tau_{T}$, which is changed to have a different dose level as MTD. Full results including scenario 2, where the MTD is at level 5, are grouped together in the summary Table 3. We recall that priors for the dose‐finding methods were set, when possible, to be informative for scenario 1, and were then kept the same for all other scenarios where they were “wrong” with respect to simulated values. Priors for PKLIM could be considered informative for all the trials. We tried using noninformative priors and achieved similar results, which is not unexpected since the response was essentially a linear regression. For that reason we do not show the results obtained with other noninformative priors for PKLIM.

In scenario 1, shown in Fig. 2A, all the methods, except for PKCRM${}_{L=7.05}$, achieved a PCS between 50% and 60%. PKCRM${}_{L=18.1}$ reached the MTD 60% of times and thus had the largest probability of finding the real MTD. Decreasing *L* down to the real value of τ used in the simulation, PKCRM achieved lower PCS but with reduced percentages of overdosing and median number of DLTs (Table 3). With L<τ PKCRM was not able to target the correct MTD, which we expected due to a strong PK constraint. PKCOV and DTOX showed similar behavior, with PCS of 55% while PKLOGIT and PKTOX had the lowest maximum number of DLTs. On average, 5 to 6 DLTs were encountered within each simulated trial of 30 subjects (20%), regardless of the method used. The minimum number of DLTs was 1 for all methods, and the maximum number of DLTs ranged from 9 to 12, showing that the choice of the dose‐finding method did not have much impact on the expected number of toxicities. In the third scenario in which the MTD is at the highest dose level, seen in Fig. 2B, the best PCS was achieved by PKCOV but at the cost of an increased maximum number of DLTs; 11 instead of 7–9 as the other methods. Within the PKCRM family, only PKCRM with the right *L* was able to identify the correct MTD in the majority of simulated trials (54%), a percentage similar to those for PKLOGIT and PKTOX (respectively 58% and 56%). PKPOP and DTOX had comparable results with PCS of 66%. Since all methods are Bayesian, we decided to apply them at least at the end of the trial even if there were no patients showing toxicity, in order to evaluate the strength of prior information.

In the next two scenarios (4 and 5, middle plots in Fig. 2), we introduced variability in the relationship between AUC and toxicity through inter‐individual variability in α. The toxicity‐AUC curve over the population is then no longer represented by the Heaviside function, i.e. a step function, but affected by individual subject sensitivity to the drug. Fig. 2C shows the results for scenario 4, which has the largest variability in α. The percentage of correct predictions decreases for all methods, because of the added inter‐individual variability. PKCOV, PKPOP and DTOX showed similar behavior with, respectively, PCS of 22%, 26% and 27%. Conversely, adjacent dose levels to the MTD were allocated more often, showing evidence of dose redistribution, as can be expected in this setting. PKTOX and PKLOGIT were less affected, with the probability of correct predictions decreasing only to 38%. The method performing best with regards to selecting the MTD was PKCRM${}_{L=7.05}$ (PCS of 54%), which also had the lower percentage of overdosing. With less inter‐individual variability in the PK‐toxicity relationship, as in scenario 5, the differences between the methods were reduced as shown in Fig. 2D. All the methods exhibited a PCS rate between 31% and 44%. PKTOX and PKLOGIT performed the worst, while the PKCRM family had the highest PCS. Again, the PKCRM methods with *L* lower than τ selected doses around the MTD more often.

In the final two scenarios (6 and 7, lower plots in Fig. 2), we investigated the impact of the inter‐individual variability in PK, with (scenario 7) and without (scenario 6) variability in α. In these two scenarios, we decreased the inter‐individual variability of clearance and volume of distribution from 70%, the value in the original data, to 30%. The results for scenario 6 are shown in Fig. 2E, and are qualitatively similar to those of scenario 1, but the percentage of correct selection now increases to values between 67% and 82%, except again PKCRM${}_{L=7.05}$. PKCRM${}_{L=10.96}$, with *L* equals to τ, has the lowest percentage of overdosing (see Table 5 in Appendix) followed by PKTOX and PKLOGIT. As before, introducing variability in α (scenario 7, Fig. 2F) decreases the percentage of correct selection for all methods. The PKCRM family achieved rates for the PCS between 43% and 56%, while all the other methods give rates between 30% and 33%.

**Figure 3:**
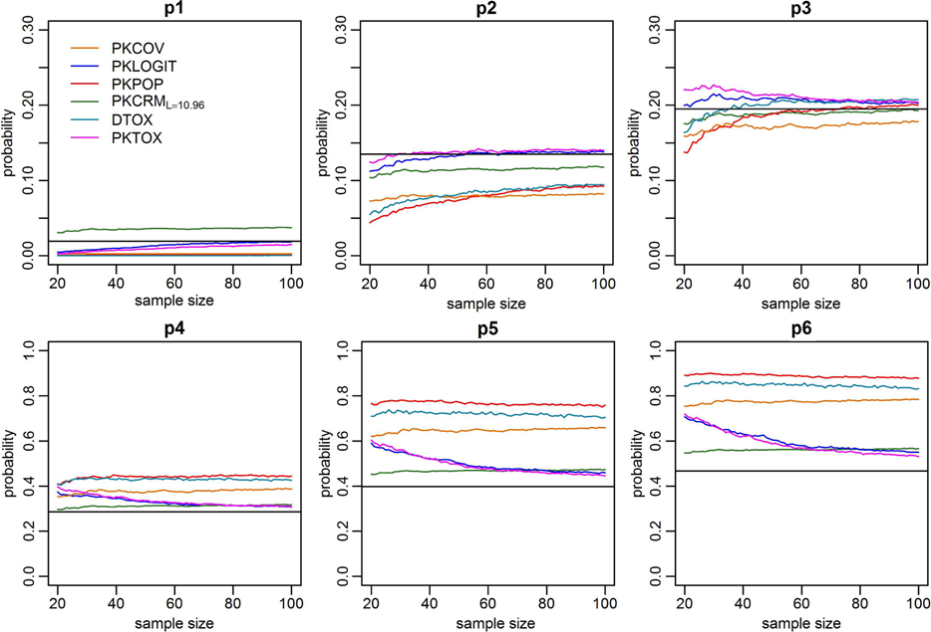
Plot of medians of estimated probabilities of toxicities for each dose versus sample size for scenario 7. p1, p2, p3, p4, p5, p6 are the probabilities of toxicity related to dose level 1, dose level 2, dose level 3, dose level 4, dose level 5, and dose level 6, respectively. The black horizontal lines represent the true probability used in the simulation, and each curve represents the median over 1000 simulations of the corresponding estimated probability for one method. Only one member of PKCRM family is shown since they have similar results.

**Figure 4:**
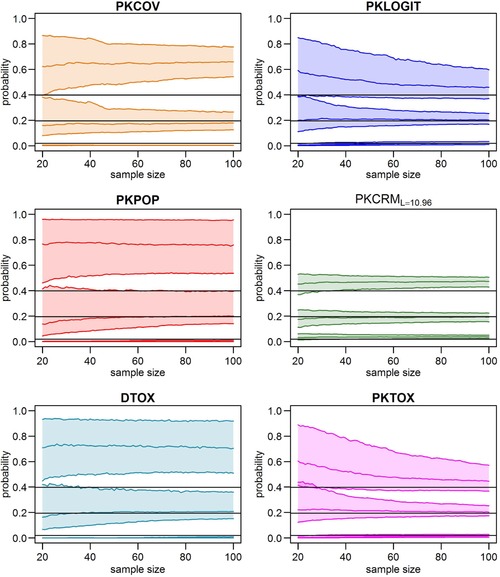
Plot of three estimated probabilities (p1, p3, and p5) of toxicity versus sample size for scenario 7 for all the methods. Each quadrant shows the three estimated probabilities using a method. Median, first, and third quartile over 1000 simulations of the corresponding estimated probabilities are plotted. The black horizontal lines represent the true probability. From below, the first is for p1, the second for p3 and the last for p5. As in Fig. 3 only one member of PKCRM family is shown since they have similar results.

We also investigated the ability of each method to estimate the dose‐toxicity relationship by considering the estimated probability of toxicity for each tested dose. This was evaluated for sample sizes increasing from 20 to 100 in order to evaluate the convergence of each method. The results are displayed in Fig. 3 for scenario 7. The black horizontal lines represent the true probability used in the simulation, and each curve represents the median over 1000 simulations of the corresponding estimated probability for one method. We plotted only one member of PKCRM family since they have similar results. Because overlaying prediction bands would make the plots illegible, we did not include them. Figure 3 shows that all the methods are able to accurately estimate the probability of toxicity of the MTD, consistent with the previous results on PCS. Also the probability of toxicity at doses adjacent to the MTD are well estimated, since, in our results true probability of toxicity is between the first and the third quartile of the distribution of estimated probability. However, only PKTOX and PKLOGIT are able to extend accurate estimation to the other dose levels. These two methods are indeed able to have a good estimation of *p*_1_ (the probability of toxicity related to dose level 1) starting from a sample size of 25 patients, and of *p*_6_ (the probability of toxicity related to dose level 6) from around 40 patients. Figure 4 shows the interquartile range for all methods for *p*_1_, *p*_3_, and *p*_5_, which are, respectively, the probabilities of toxicity related to dose level 1, 3, and 5.

Similar behavior was also observed for all other scenarios (plots not shown). PKTOX and PKLOGIT are able to give good estimates in each scenario, while other methods tend to estimate the probabilities of toxicity correctly only for the MTD and adjacent doses. The PKCRM family is able to estimate all probabilities of toxicity when the skeleton is identical to the real probability of toxicity. Over all scenarios, we noted that DTOX, PKCOV, and PKPOP gave more accurate estimates for extreme probabilities in scenarios in which $\omega_{\alpha }$ is equal to zero.

Finally, we checked estimation of the PK parameters at the end of the trial for each method. We used the simulated data and fitted models with Monolix. Despite the diverse distributions in dose allocations, all the results were similar suggesting that the choice of the method does not influence parameter estimation.

## 5 Discussion

In the present study, we reviewed, extended, and developed methods that take into account PK measurements in sequential Bayesian adaptive designs for early dose‐finding studies in small populations. We adapted existing methods for the same clinical setting, which was taken from a real PK/PD example, the development of the TGF‐β inhibitor LY2157299 in patients with glioma (Gueorguieva et al., 2014), where toxicity occurs after a high exposure to the drug. The same example has been used by Lestini et al. (2015) to evaluate adaptive designs in model‐based approaches. We simulated a range of scenarios reflecting different assumptions and different amounts of inter‐individual variability, in order to assess the performance of each method in term of the maximum tolerated dose as well as the estimation of the probabilities of toxicity associated with each candidate dose. With a fixed threshold in the AUC‐toxicity relationship, the occurrence of toxicity assumed to occur whenever the exposure exceeds this threshold. This can reflect the physician's recommendation on a target concentration not to be exceeded though it may be too deterministic to be plausible in practice, as it reflects a strong clinical constraint set for safety reasons when nonmonitorable toxicity has been observed in preclinical phases during drug development. Introducing a variability in the AUC‐toxicity relationship allowed us to simulate a more complex but also more physiologically reasonable situation in which the sensitivity to drug concentrations varies across subjects.

The two main findings from the simulated clinical trials are first, that having good prior information on PK, from preclinical data or other previous clinical trials, can help in decreasing overdosing without changing the PCS, and second, that only methods that estimate the probability of toxicity through the dose‐AUC and AUC‐tox relationships are able to estimate the dose‐toxicity relationship adequately.

The first method presented, PKCOV, can be viewed as a “continuous” CRM with a PK covariate added. It resulted in one of the least accurate models. For instance, in scenario 4, following the PKCOV method, the wrong MTD was chosen in 77% of cases and adjacent dose levels to the MTD were allocated more often than the correct dose. A similar situation could be found in scenario 2. Adding the patient's AUC distance from the mean estimated value as a covariate can be seen as a way to take into account a measure of interindividual variability. In effect $\Delta {z_{d}}_{k}$ depends on $\omega_{CL}$ since, after *r* patients allocated at dose *d*, it follows a normal distribution with mean equal to zero and variance equal to $\left(r+1\right)\omega_{CL}^{2}/r$. It does not depend on dose, therefore the covariates in model 1 are not correlated.

The PKCRM methods were developed as combining the PKLIM, with the standard CRM. When PKLIM is used alone, we found it had reasonable performance since, essentially, it is a linear regression model resulting from a linear compartment model used to analyze the PK data, but as it does not incorporate the observed toxicities, it led to more patients showing DLTs (data not shown). Another issue was that the dose allocation of PKLIM is driven only by the constant threshold *L*. As the method does not take into account an adaptive updating of this threshold, a wrong initial guess can lead to poor results. For that reason, the method we present in this paper is PKCRM, which couples PKLIM and CRM: in that way, PKLIM can take the role of a second constraint based on AUC. Nonetheless, even in this model the choice of *L* is crucial. As shown in the tables in the Results section, when *L* is equal to the real toxicity threshold $\tau_{T}$, PKCRM maintained, more or less, the performance of CRM while reducing the risk of overdosing. When *L* is greater than $\tau_{T}$, the more we increase *L*, the nearer we get to the unmodified CRM (PKCRM${}_{L=18.1}$ in our examples) as we relax the PK constraint. Conversely, when *L* is lower than $\tau_{T}$, as we expected, PKCRM was not able to achieve the real MTD since PK constraint drives the dose selection downwards. For that reason careful choice of *L* is necessary before starting trials: it could either improve considerably the PCS (scenario 6) or lead to very wrong results, which may make it difficult to use in practice when little information is available beforehand. In the motivating example used in our simulations, a threshold for the AUC was derived from animal studies (Gueorguieva et al., 2014), which is common practice in drug development. Approaches to setting the threshold range from empirical (extrapolating a nontoxic AUC to humans and dividing by 10 for safety) to heavily mechanistically based (using predictions from, e.g. PBPK models). An alternative in practice could be to test different thresholds, to adapt the thresholds during the trials based on the first cohorts included in the trial, or to combine the results obtained with different thresholds through model averaging approaches.

Following this idea, we can see PKLOGIT as an attempt to adaptively estimate the threshold *L* of PKLIM. This requires an underlying model, mechanistically increasing the number of equations and parameters. This could lead to difficulties in parameter estimation when the relationship between toxicity and AUC is represented by an Heaviside function, as in four of our Scenarios (1–3,6). However, when the MTD is not located at the last dose level, the logistic function can have enough data to approximate, more or less, the step function. Together with PKTOX this method had the best ability to estimate the probabilities of toxicity over the range of candidate doses used in the study, suggesting that it provides us with more information on the dose‐concentration‐toxicity relationships than the previous methods considered. The two equations work at different levels: the model that links $p_{T}$ and log (AUC) operates at the patient level and the Gaussian regression operates at the population level. Computing $P(y_{i+1}=1|\beta ={\hat{\beta }}_{i})$ involves an integral and is likely to be affected by the errors from both regressions, thus in some cases they could interact to contribute a bigger final error, especially in a small sample setting. We tried to add a more simple population level step in PKPOP similar to a logistic regression relating $p_{T}$ to log(d), but in that case the regression is made at the AUC population level (AUC =d/CL), that is part of the variability is already estimated in a previous model. From the results one can see that this simpler model works better in cases when toxicity versus AUC can be modeled by a step function, especially in scenario 2–3. It should be noted however, that it converges to $p_{T}$ versus log(d) more slowly, needing larger numbers of patients to be recruited into the trial.

In the last two methods, DTOX and PKTOX, we investigate two different ways of estimating the dose‐toxicity curve. For that reason, both approaches use the real link function (Φ), even if, after simulations, the logit link had the same performance of the probit link. The difference between the two methods is that DTOX directly models the toxicity as a function of dose while PKTOX uses drug exposure, with an increased number of equations and parameters. PKTOX, like PKLOGIT, is therefore more of a AUC targeting method than a dose targeting method. This suggests that it could be useful for a personalized medicine approach targeting individual drug exposure. It would also reflect the pharmacokinetics of the drug in a more subtle way than considering only the dose, as different dosing regimens could lead to different drug exposures for nonlinear pharmacokinetics, and AUC can then be predicted from the PK model. It is useful to recall that DTOX and PKCRM${}_{L=18.1}$, which approaches the unmodified CRM for all scenarios except Scenario 3, are two methods that do not use PK information. They are able to well estimate the probability of toxicity linked to the MTD, but not all the other probabilities of toxicity, as we showed in Figs. 3 and  4 (in those figures PKCRM${}_{L=10.96}$ only is plotted, but all PKCRM method behaved in a similar way). These systematic deviations can be attributed to the usual dose allocation process, that tends to allocate the majority of the patients near to the MTD. Therefore, the methods can get information in this limited range, that is, the MTD and adjacent doses, but do not gain information on other doses. This leads to a very good estimation of only a limited part of the dose‐toxicity curve so that increasing the sample size does not improve estimation of the other parts of this curve for these methods.

Dose‐finding methods can be broadly classified according to two different types of approaches, the “dose‐estimators” and the “dose‐finders” (Rosenberger and Haines, 2002). In the first type, the aim is to estimate the entire dose‐toxicity curve, and to determine the dose associated with a given percentile of the dose‐toxicity curve. Model‐based approaches combined with optimal design belong to this framework (Bornkamp et al., 2007). The second type, which is the approach usually applied in the oncology setting, the aim is to home in on the maximum tolerated dose amongst the doses tested in the trial, without estimating the entire dose‐toxicity curve (O'Quigley et al., 1990; Whitehead and Brunier, 1995; Babb et al., 1998), so that results are valid only for the doses tested. The CRM, since it is built directly to take care of a discrete number of doses, is a “dose‐finder” method. After choosing the skeleton, the regression makes sense only for the given set of the doses and it is meaningless to extrapolate to other doses after the estimation. For that reason, PKCRM${}_{L=18.1}$, which essentially tends to the unmodified CRM, had good performance in PCS, except in the last scenario, in which the CRM skeleton played an important role. It should be noted that the same skeleton was used across all scenarios, which reflects the approach used in practice where the skeleton could be based on prior information. On the other hand, all the other methods are more “dose‐estimator” methods, since they try to estimate the entire dose‐toxicity curve, and only in the last step of the method do they focus on the discrete set of candidate doses. In that case, extrapolation to the entire dose‐toxicity curve is a natural extension. Our results show that dose‐finder approaches home in on the MTD in an efficient way, but that the probability of toxicity was estimated poorly for doses far from the MTD. Dose‐estimators on the other hand allowed us to better estimate the probabilities of toxicities throughout the range of doses, which make them more useful when considering the entire drug development process to integrate the information collected throughout successive trials. Dose‐finders, on the other hand, focus on determining the probability of toxicity for each dose level and then select the one near to the target, so that extrapolation to different dose ranges is difficult.

Although our results partly depend on the settings of our simulation study, they were consistent across different simulation scenarios including variability in the patient sensitivity. They should hold regardless of the actual underlying PK relationship as long as the link between the exposure and the occurrence of toxicity is reasonably well accounted for. The actual benefit of including PK may vary, as shown for instance in the first scenario where the priors and working models are already well informed so that the PK information does not bring much added information. It would also be worth investigating the impact of departures from the model assumptions, such as the presence of nonlinear pharmacokinetics, although such models could be accommodated within the proposed methods by changing the regression submodels. However, our simulations show that taking account of the exposure does not harm the determination of the MTD but provides more precise estimation of the entire dose‐toxicity relationship. Further work is ongoing to evaluate methods taking into account both PK and PD in order to target effective doses.

In conclusion, our results show that dose‐estimators can provide more information on the dose‐toxicity relationship and the role of exposure, while traditional methods are more specifically targeted toward finding the MTD and will derive it more efficiently. Studies have shown that the wealth of data accumulated during clinical development is insufficiently utilized, and suggested analysis, modeling, and simulation can capitalize sequentially on accrued data to streamline drug development and provide better medicines to better targeted patients (Arrowsmith, 2011). The dose‐estimator methods require additional PK information, but the models involved are simpler than in full‐model based approaches and can easily be implemented in practice. Ultimately, there is a trade‐off between immediate gain, which is the determination of the MTD for the next study, and long‐term goals, that is, accruing information about the entire dose‐toxicity relationship. We would argue that dose‐estimators can also be useful in the long run to extrapolate conclusions to different dosage regimens and ranges of doses.

## Conflict of interest

*The authors have declared no conflict of interest*.

## Supporting information

Supporting Information.
